# Fine-Grained and Flexible Dual Authentication for IoT-Connected Healthcare Sensor Networks

**DOI:** 10.3390/s26165223

**Published:** 2026-08-18

**Authors:** Huiying Hou, Jianyu Miao, Yucong Ma, Xuerui Gan, Xuefeng Li

**Affiliations:** 1 The Key Laboratory of Grain Information Processing and Control, Henan University of Technology, Zhengzhou 450001, China; 2 The School of Artificial Intelligence and Big Data, Henan University of Technology, Zhengzhou 450001, China; jymiao@haut.edu.cn (J.M.); ycma@stu.haut.edu.cn (Y.M.); or xf_li@haut.edu.cn (X.L.); 3 School of Electrical Engineering, Henan University of Technology, Zhengzhou 450001, China; xueruigan@stu.haut.edu.cn

**Keywords:** healthcare sensor networks, Internet of Medical Things, wearable sensors, dual authentication, attribute-based sanitizable signature, privacy-preserving authentication, outsourced verification

## Abstract

IoT-connected healthcare sensor networks require authenticated and privacy-preserving data exchange among wearable sensors, mobile medical terminals, edge gateways, cloud servers, and medical institutions. Existing authentication schemes for healthcare IoT often bind signatures directly to user identities, exposing sensitive personal or institutional information and imposing heavy verification costs on resource-constrained sensing devices. To address this problem, we propose a fine-grained and flexible dual authentication scheme for healthcare sensor networks. In the proposed scheme, health data and diagnoses are signed with a fresh signing key and a fine-grained access control policy each time, so that the signer identity remains hidden while authorized entities can still modify permitted parts of signed data. No entity other than an authorized entity can trace a malicious signer or modify signed data without changing the data source. To support lightweight verification in sensor-edge-cloud deployments, we further present a verifiable outsourced authentication scheme that outsources time-consuming pairing operations to cloud servers; the online verification process then requires only six multiplication operations. As a fundamental technical component, we present a practical attribute-based sanitizable signature with shorter signature and key lengths and more efficient signing and signature-changing operations than the state-of-the-art policy-based sanitizable signature (P3S). Formal security analysis and experiments demonstrate the security and practicality of the proposed scheme for privacy-preserving healthcare sensing and medical data exchange.

## 1. Introduction

Healthcare sensor networks are increasingly built from wearable sensors, mobile medical terminals, home gateways, edge devices, cloud services, and hospital information systems. These connected sensing environments enable continuous physiological monitoring, teleconsultation, emergency triage, and data-driven diagnosis, but they also enlarge the attack surface: health data and diagnosis messages may traverse heterogeneous sensing devices, edge gateways, cloud servers, and multiple medical institutions before reaching the final verifier. Therefore, authentication mechanisms for IoT-connected healthcare must protect both data integrity and personal privacy while remaining efficient enough for resource-constrained sensors, gateways, and mobile medical devices.

**Figure 1:**
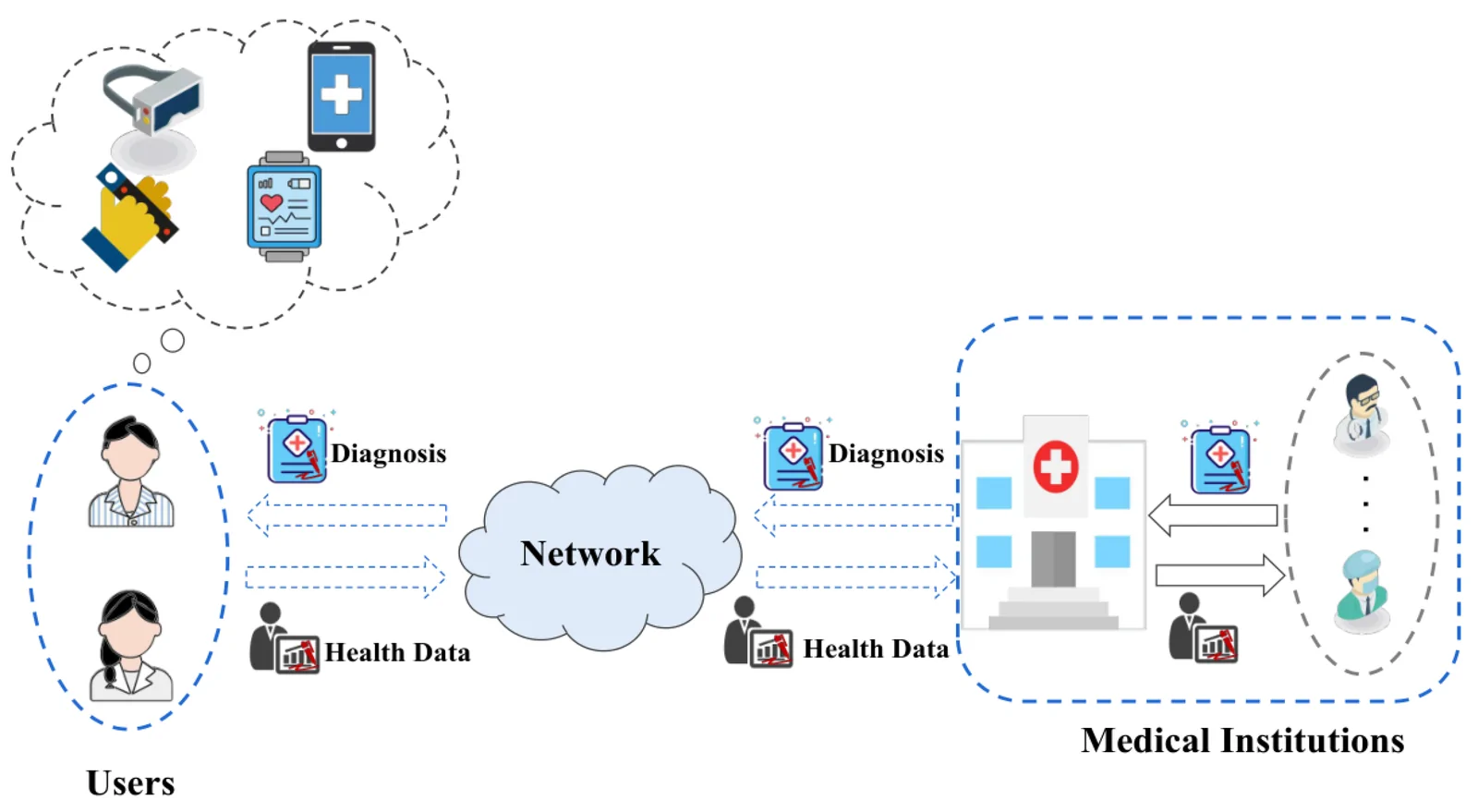
The structure of IoT-connected healthcare sensor networks. The figure shows how wearable sensors collect health data, how personal identifiers are removed before transmission, how gateways and edge/cloud nodes relay signed data, and how authorized medical institutions recover the necessary medical context for diagnosis.

The Internet of Things (IoT) is dramatically changing the way we live through a variety of applications such as smart cities, smart transportation and connected healthcare [1]. As a vital application of IoT, IoT-connected healthcare is proposed to improve the efficiency of health systems and reduce health costs [2]. Users can monitor their health status in real-time and enjoy teleconsultation with web-connected wearable devices. IoT-connected healthcare allows users to obtain diagnoses from medical institutions anytime and anywhere, which provides certain protection for residents’ health. Despite the aforementioned advantages, IoT-connected healthcare poses numerous security challenges. IoT-connected healthcare is considered an on-demand service, i.e., it provides health services only to users who subscribe to and pay for it. Therefore, the medical institutions need to verify the validity of the user’s identity after receiving the request. In most of the existing authentication schemes for IoT-connected healthcare, users need to sign the health data with a signing key bound to their identity. This exposes sensitive user identity information. Moreover, such schemes only consider the authentication of the user. In reality, however, authenticating the source of data for remote diagnosis is just as important. Only a diagnosis from a legitimate medical institution is credible. In some cases, the source of the diagnosis also reveals the patient’s privacy to some extent. For instance, the adversary could infer that the patient might suffer from reproductive diseases based on the diagnosis from a reproductive surgeon. Normally, a patient with a reproductive disease does not want other people to know about it. The rapid development of IoT-connected healthcare is making this an increasingly common problem. Therefore, the implementation of dual authentication for privacy protection in IoT healthcare networks is a very important problem. Signing the data with a pseudonym before uploading it to the network is one of the potential solutions to the above problem. The health data, such as Health Level Seven (HL7) [3], contains the identifier, gender and birthdate of the user. As shown in Figure 1, a user signs the health data, from which the personal information has been removed, with a pseudonym. Then, the user uploads the signed data to the network. Using only one or a few pseudonyms cannot realize the privacy preservation of the user’s identity. Therefore, the user needs to save numerous pseudonyms in advance, but it causes a serious storage burden. After receiving the signed health data, the medical institutions first verify whether the user is valid. In order to authenticate users, medical institutions need to pre-store the mapping between all possible pseudonyms and all user identities they serve. Clearly, such an approach is impractical. This is because the number of users to be served is extremely large. Another problem caused by this solution is that medical institutions are unable to make an accurate diagnosis according to the health data, which includes none of the user’s personal information, such as age, gender, etc. As shown in Figure 1, medical institutions need to sign their diagnosis with pseudonyms and then send the signed diagnosis to users. In the same vein, users need to pre-store the mappings between all possible pseudonyms and all candidate medical institutions’ identities. This imposes a heavy storage burden on resource-constrained users that far exceeds their capacities. This results in the user being unable to authenticate the medical institutions exactly. The adversary may successfully pretend to be a legitimate medical institution and give a malicious diagnosis, which may endanger the user’s life. Moreover, in reality, the diagnosis is not only accessible to the user, but also to authorized family members and friends. Furthermore, the health data should be accessed by multiple authorized medical institutions for better treatment. Considering the above limitations, three questions naturally arise:

- Is there any approach that enables the signature to expose no information about the signer’s identity while the signer does not need to save numerous pseudonyms?
- Does any method exist to allow someone other than the signer to modify the signed data non-interactively without changing the data source?
- Does any method exist to allow the signer to specify who can modify the signed data and which parts of the data can be modified through a fine-grained access control policy?

**Our Contribution.** In order to address the above problems in IoT-connected healthcare sensor networks, we propose a fine-grained and flexible dual authentication scheme that can be deployed over wearable-sensor, mobile-health, and edge-assisted medical communication networks. The scheme treats sensor gateways, edge nodes, and mobile medical terminals as potentially resource-constrained communication and verification participants: they may forward, verify, or help process authenticated healthcare data, but they should not learn users’ or medical institutions’ private identities. Considering that healthcare sensors and edge gateways have limited computation and energy budgets, we further design a verifiable outsourced authentication scheme that outsources the time-consuming pairing operations to two mutually distrusted cloud servers.

In addition, to realize a fine-grained privacy-preserving dual authentication scheme for IoT-connected healthcare sensor networks, we propose a new attribute-based sanitizable signature (ABSS). Our main contributions are summarized as follows:

- **Fine-grained Access Control:** Users and medical institutions can specify who can modify the data and which parts of the data they sign can be modified through a fine-grained access control policy. Only authorized entities can modify the permitted parts of the signed data.
- **Privacy Preserving:** In our scheme, to hide the identity, the user and the medical institutions sign the data with a fresh related signing key each time, which belongs to the same equivalence class. Unauthorized entities without the corresponding trapdoor cannot link these different keys, i.e., unauthorized entities cannot trace the original signer and modifier of the signed data. Thus, the transmitted data do not reveal the user’s personal information.
- **Transparency:** An authorized entity can sign the modified data in a non-interactive way, and the signature is difficult to distinguish from the original signature.
- **Accountability:** Neither the original signer (the user or the medical institutions) nor the authorized modifier can accuse the other’s signature. Only authorized entities can trace the malicious signer or modifier accurately.
- **Friendly to Resource-Constrained Sensors and IoT Nodes:** We further design a verifiable outsourced authentication scheme for IoT-connected healthcare sensor networks. In this scheme, the time-consuming pairing operations are outsourced to two cloud servers that are mutually distrusted. In this way, the verification process only needs six multiplication operations, which is friendly to resource-constrained users, wearable devices, sensor gateways, and edge relay nodes.

In addition, the signed data contains a timestamp to ensure accurate treatment and prevent replay attacks. On the theoretical side, we present a new ABSS based on Ciphertext-Policy Attribute-Based Encryption (CP-ABE) and Signature with Flexible Public Key (SFPK). In ABSS, the original signer can specify the modifiable part of the data and the user who can modify the signed data through a fine-grained access control policy. ABSS supports the privacy preservation of the signer’s identity. Only authorized users can trace signers and modifiers of signed data and modify the parts of the data that are allowed to be modified. Encouragingly, ABSS has shorter signature and key lengths and more efficient signing and signature-changing operations than the most advanced policy-based sanitizable signature (P3S) [4], as shown in Table 1.

### 1.1. Related Work

The first sanitizable signature scheme was proposed by Ateniese et al. [5] in 2005. However, this scheme did not provide the complete definition and security proof of the sanitizable signature. Next, Brzuska et al. [6] solved the above problems. Canard et al. [7] introduced the definition and general construction of a trapdoor sanitizable signature. Then, the definition of another accountable trapdoor sanitizable signature was proposed by Lai et al. [8], which is based on the chameleon hash. However, the above two schemes did not give the detailed parameters for a sanitizable signature scheme. Then, many sanitizable signature schemes were presented [9,10]. Firstly, we want to address the problem of privacy-preserving dual authentication for IoT-connected healthcare by using one of the above sanitizable signature schemes. Unfortunately, none of the above schemes are suitable for IoT-healthcare because none of them support fine-grained control of candidate sanitizers and accountability for malicious users. Attribute-based cryptography schemes [11,12,13,14,15] can help solve the above problem. The ABSs proposed by Maji et al. [14] and Li et al. [16] achieve security under the generic group model and security under the random prediction model, respectively. However, none of the above ABSs supports an expressive access structure. The non-monotone access structure supports expressive access. Based on this property, Okamoto and Takashima [17] proposed an ABS scheme that exploits a non-monotonic access structure. Next, several ABS schemes were proposed [18,19,20]. None of the users in the above ABS schemes can modify the data and update the signature in the name of the data owner. Sanitizable signature schemes [4,21,22,23] can solve the problem. The scheme in [21] did not introduce attribute-based sanitizable signatures in detail, while the scheme proposed in [22] did not support an expressive access structure. The scheme proposed by [23] did not achieve traceability of the signer. In the scheme in [4], the number of signed data blocks is fixed, and the inadmissible block set needs to be saved. In a real IoT-connected healthcare environment, the identities of users and medical institutions should be fully protected.

Recent studies on IoT healthcare authentication further confirm that lightweight verification, privacy protection, and resistance to practical attacks are central requirements for medical sensor networks [24,25,26,27]. These studies mainly focus on authenticating devices, sessions, or data dissemination in healthcare IoT, while attribute-based signatures and sanitizable signatures mainly focus on expressive authorization and controlled data modification. The remaining gap is that a healthcare sensor-network scheme should simultaneously support dual authentication, signer-identity hiding, fine-grained authorization of modifiable data blocks, accountability of malicious signers or modifiers, and lightweight verification for resource-constrained sensors and gateways. The proposed ABSS differs from P3S because it replaces identity-bound signing/public-key use with fresh SFPK-related signing keys and uses CP-ABE to release the signing capability only to entities whose attributes satisfy the access policy. Therefore, CP-ABE is adapted as the fine-grained authorization layer, SFPK is adapted as the unlinkable fresh-key layer, and their integration into a sanitizable dual-authentication workflow with outsourced verification is the new component of this paper.

However, none of the above schemes can realize the privacy preservation of the signer’s identity in a setting where healthcare data may be forwarded through wearable sensors, IoT devices, edge nodes, cloud services, and medical institutions. This paper proposes a fine-grained privacy-preserving dual authentication scheme to secure users’ personal information in IoT-connected healthcare sensor networks. Based on Ciphertext-Policy Attribute-Based Encryption (CP-ABE) and Signature with Flexible Public Key (SFPK), an attribute-based sanitizable signature is designed.

### 1.2. Organization

This paper is organized as follows. We concisely review the fundamental knowledge related to this paper in Section 2. In Section 3, we give the system model and security model definitions. In Section 4, we characterize the proposed scheme. In Section 5, we analyze the security and performance of the proposed scheme. Finally, we conclude the paper in Section 6.

## 2. Materials and Methods

In addition to common cryptographic tools, this paper uses Prime Order Bilinear Groups, Monotone Span Program, Ciphertext-Policy Attribute-Based Encryption ( CP-ABE), Non-Interactive Zero-Knowledge Proof (NIZK), Signatures with Flexible Public Keys (SFPK), and Programmable Hash Functions (PHF) as the main technical preliminaries for constructing the proposed authentication scheme.

### 2.1. Prime Order Bilinear Groups

Let $\Phi (1^{\lambda })$ be a function with security parameter λ as input and output a tuple

$$(p,G,G_{1},G_{T},\hat{e}),$$

where *G*, $G_{1}$, and $G_{T}$ are three multiplicative cyclic groups with prime order *p*. A mapping

$$\hat{e}:G\times G_{1}\to G_{T}$$

is called a bilinear mapping if it satisfies the following properties:

- **Bilinearity:** For all u∈G, $v\in G_{1}$, and $a,b\in Z_{p}$,
  $$\hat{e}\left(u^{a},v^{b}\right)=\hat{e}\left(u^{b},v^{a}\right)=\hat{e}{\left(u,v\right)}^{ab}.$$
- **Non-degeneracy:** For all u∈G and $v\in G_{1}$, $\hat{e}\left(u,v\right)\neq 1$.
- **Computability:** For all u∈G and $v\in G_{1}$, $\hat{e}\left(u,v\right)$ can be efficiently computed.

### 2.2. Monotone Span Program

A monotone span program can be represented by a triad M=(F,M,f), where *F* is a field, *M* is an a×b matrix, and

$$f:\left\{1,\ldots ,a\right\}\to \left\{p_{1},\ldots ,p_{n}\right\}.$$

The elements of the matrix *M* are all taken from the field *F*. The sub-matrix $M_{B}$ with $B\subseteq \{p_{1},\ldots ,p_{n}\}$ is accepted if the rows of $M_{B}$ span the vector (1,0,…,0).

### 2.3. Ciphertext-Policy Attribute-Based Encryption

#### Definition: Ciphertext-Policy Attribute-Based Encryption (CP-ABE)

The CP-ABE primitive consists of the following four algorithms:

- $\mathrm{CP}\text{-}\mathrm{ABE}.\mathrm{Setup}(1^{\lambda })$ takes the security parameter λ as input and outputs the public parameter $pp_{\mathrm{CP}\text{-}\mathrm{ABE}}$ and the master secret key $msk_{\mathrm{CP}\text{-}\mathrm{ABE}}$.
- $\mathrm{CP}\text{-}\mathrm{ABE}.\mathrm{Encrypt}(pp_{\mathrm{CP}\text{-}\mathrm{ABE}},A,m)$ takes the public parameter $pp_{\mathrm{CP}\text{-}\mathrm{ABE}}$, the access policy *A*, and the message *m* as input, and outputs the ciphertext $C_{\mathrm{CP}\text{-}\mathrm{ABE}}$.
- $\mathrm{CP}\text{-}\mathrm{ABE}.\mathrm{KeyGen}(msk_{\mathrm{CP}\text{-}\mathrm{ABE}},S)$ takes the master secret key $msk_{\mathrm{CP}\text{-}\mathrm{ABE}}$ and the set of attributes *S* as input, and outputs the secret key $sk_{\mathrm{CP}\text{-}\mathrm{ABE}}$ for *S*.
- $\mathrm{CP}\text{-}\mathrm{ABE}.\mathrm{Decrypt}(pp_{\mathrm{CP}\text{-}\mathrm{ABE}},C_{\mathrm{CP}\text{-}\mathrm{ABE}},sk_{\mathrm{CP}\text{-}\mathrm{ABE}})$ takes the public parameter $pp_{\mathrm{CP}\text{-}\mathrm{ABE}}$, the ciphertext $C_{\mathrm{CP}\text{-}\mathrm{ABE}}$, and the secret key $sk_{\mathrm{CP}\text{-}\mathrm{ABE}}$ as input, and outputs the message $m^{\prime }$.

The detailed definition of correctness and formal security proof of CP-ABE are given in [28].

### 2.4. Non-Interactive Zero-Knowledge Proof (NIZK)

Let

L={x∣∃ω:R(x,ω)=1},

where *L* is an NP-language with associated witness relation *R*. A non-interactive proof system Ω for the language *L* consists of the following three algorithms:

- ${\mathrm{Setup}}_{\Omega }\left(1^{\lambda }\right)$ takes the security parameter λ as input and outputs the common reference string $crs_{\Omega }$.
- ${\mathrm{Prove}}_{\Omega }\left(crs_{\Omega },x,\omega \right)$ takes $crs_{\Omega }$, the statement *x*, and the corresponding witness ω as input, and outputs the proof π.
- ${\mathrm{Verify}}_{\Omega }\left(crs_{\Omega },x,\pi \right)$ takes $crs_{\Omega }$, the statement *x*, and the proof π as input, and outputs 1 if π is valid; otherwise, it outputs 0.

### 2.5. Signature with Flexible Public Key

#### Definition: Signature with Flexible Public Keys (SFPK)

This type of signature contains the following eight algorithms:

- $\mathrm{SFPK}.\mathrm{Setup}(1^{\lambda })$ inputs the security parameter λ and outputs the public reference string ρ, which is then used as the default input to subsequent algorithms.
- $\mathrm{SFPK}.\mathrm{KGen}(1^{\lambda },\omega )$ inputs the security parameter λ and the random coins ω, and outputs the public/private key pair $\left\{pk_{\mathrm{SFPK}},sk_{\mathrm{SFPK}}\right\}$.
- $\mathrm{SFPK}.\mathrm{TKGen}(1^{\lambda },\omega )$ inputs the security parameter λ and the random coins ω, and outputs the public/private key pair $\left\{pk_{\mathrm{SFPK}},sk_{\mathrm{SFPK}}\right\}$ and the trapdoor δ.
- $\mathrm{SFPK}.\mathrm{Sign}(sk_{\mathrm{SFPK}},m)$ inputs the signing key $sk_{\mathrm{SFPK}}$ and the message $m\in {\left\{0,1\right\}}^{*}$, and outputs the signature σ.
- $\mathrm{SFPK}.\mathrm{ChkRep}(\sigma ,pk_{\mathrm{SFPK}}^{\prime })$ outputs 1 if $pk_{\mathrm{SFPK}}^{\prime }\in {\left[pk_{\mathrm{SFPK}}\right]}_{R}$; otherwise, it outputs 0.
- $\mathrm{SFPK}.\mathrm{ChgPK}(pk_{\mathrm{SFPK}},r)$ inputs the public key $pk_{\mathrm{SFPK}}$, which is a delegate of the equivalence class ${\left[pk_{\mathrm{SFPK}}\right]}_{R}$, and random coins *r*, and outputs a different delegate $pk_{\mathrm{SFPK}}^{\prime }\in {\left[pk_{\mathrm{SFPK}}\right]}_{R}$.
- $\mathrm{SFPK}.\mathrm{ChgSK}(sk_{\mathrm{SFPK}},r)$ inputs the secret key $sk_{\mathrm{SFPK}}$, which is a delegate of the equivalence class ${\left[sk_{\mathrm{SFPK}}\right]}_{R}$, and random coins *r*, and outputs a different delegate $sk^{\prime }\in {\left[sk_{\mathrm{SFPK}}\right]}_{R}$.
- $\mathrm{SFPK}.\mathrm{Verify}(pk_{\mathrm{SFPK}},m,\sigma )$ inputs the public key $pk_{\mathrm{SFPK}}$, the message *m*, and the signature σ, and outputs 1 if the signature is valid; otherwise, it outputs 0.

The scheme [26] defines the correctness and a formal security proof of SFPK in detail.

### 2.6. Programmable Hash Functions

A programmable hash function (PHF) [27] consists of the following two PPT algorithms:

- $K_{\mathrm{PHF}}\leftarrow \mathrm{PHF}.\mathrm{Gen}\left(1^{\lambda }\right)$ inputs the security parameter λ and outputs the key $K_{\mathrm{PHF}}$.
- $y\in G_{1}\leftarrow \mathrm{PHF}.\mathrm{Eval}\left(K_{\mathrm{PHF}},X\right)$ inputs the key $K_{\mathrm{PHF}}$ and $X\in {\left\{0,1\right\}}^{𝓁(\lambda )}$, and deterministically outputs the hash value *y*, where 𝓁(λ) represents the length of λ.

## 3. System Model and Security Model

### 3.1. System Model

**Figure 2:**
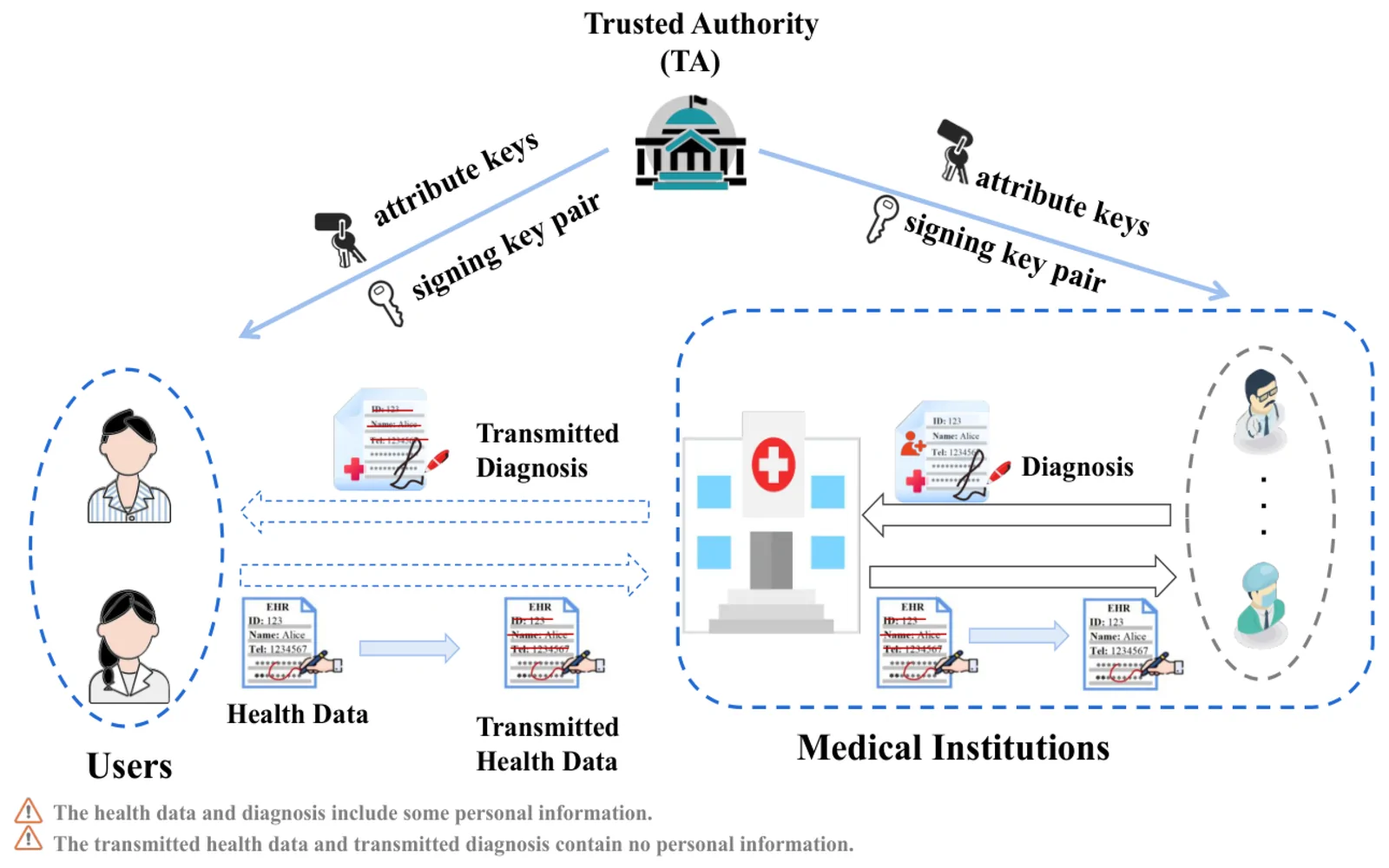
The IoT-connected healthcare sensor-network system model. The model separates the roles of users, wearable sensors, sensor gateways/edge relays, cloud servers, the trusted authority, and medical institutions, and highlights which entities may forward data, verify signatures, modify authorized fields, or trace malicious behavior.

As shown in Figure 2, the system model of the proposed scheme is organized for an IoT-connected healthcare sensor-network scenario and contains four types of entities: users, sensor/edge relay nodes, the trusted authority (TA), and medical institutions. The same cryptographic framework can also be deployed in other mobile IoT systems with similar security requirements.

- **Users:** Users collect health data through wearable or portable IoT devices. A user may be malicious in the sense that an unauthorized user may attempt to impersonate a legitimate subscriber of a remote diagnosis service. Multiple IoT devices owned by the same user correspond to the same set of keys. Before transmission, the user removes personal information from the collected health data, specifies which medical institutions may modify the signed data and which parts may be modified, and signs the transmitted health data with a fresh related signing key.
- **Sensor/edge relay nodes:** Wearable sensors, home gateways, mobile medical terminals, and edge relay nodes provide sensing, communication, relay, and lightweight verification support between users and medical institutions, especially in continuous monitoring, emergency response, rural coverage, temporary medical service, and public-safety healthcare scenarios. These nodes are not assumed to be fully trusted with identity information. They may forward signed health data, help perform verification, or interact with outsourced cloud servers, but they should not be able to infer the identities of users or medical institutions from transmitted signatures.
- **Trusted Authority:** The TA is considered honest. It generates signing private keys for users and medical institutions, and issues attributes and attribute keys to all entities other than itself. The TA also supports the traceability function required for accountability.
- **Medical Institutions:** Medical institutions are semi-honest. They can add or remove users’ personal information from signed data when they are authorized by the access control policy, generate diagnoses based on the recovered medical context, and sign diagnoses with fresh related signing keys. A valid signature generated after authorized modification is indistinguishable from an original signature.

The workflow is as follows. First, a user collects health data via wearable or portable sensing devices and removes personal information to obtain transmitted health data. The user signs the transmitted health data with a fresh related signing key and a fine-grained access control policy. The signed data can then be forwarded through sensor gateways or edge relay nodes to medical institutions. Only authorized medical institutions with the corresponding trapdoor can link the user’s different signing keys and trace the user’s identity. After receiving signed health data, an authorized medical institution verifies the data source, restores necessary personal information, and generates an accurate diagnosis. The medical institution then signs the diagnosis with a fresh related signing key and specifies a new access control policy for subsequent modification. When the diagnosis is transmitted back through the healthcare sensor network, authorized medical institutions can remove the user’s personal information and generate a new indistinguishable signature. Finally, the user verifies the medical institution’s identity and accepts the diagnosis only if the verification succeeds.

### 3.2. Design Goals

To realize fine-grained and flexible privacy-preserving dual authentication for IoT-connected healthcare sensor networks, our scheme aims to achieve the following goals.

- **Fine-grained Access Control:** Users and medical institutions can determine which entities may modify the signed data and which parts of the signed data may be modified through a specified fine-grained access control policy. Authorized entities can generate a new signature for modified data in a non-interactive manner, and the new signature is indistinguishable from the original signature.
- **Privacy Preserving:** The personal information of users and medical institutions should remain hidden during transmission through sensor, IoT, edge, and cloud communication channels.
- **Transparency:** The signature of modified data generated by authorized entities is indistinguishable from the signature of the original data.
- **Accountability:** Neither the original signer nor the authorized modifier can falsely accuse the other of signing. Authorized entities can trace malicious signers or modifiers accurately.
- **Sensor/IoT Friendliness:** The authentication mechanism should reduce online verification overhead so that sensor gateways, wearable devices, mobile medical terminals, and other resource-constrained IoT entities can participate in secure healthcare data exchange.

### 3.3. Definition

#### Definition: Fine-Grained and Flexible Privacy-Preserving Dual Authentication Scheme for IoT-Connected Healthcare Sensor Networks

A fine-grained and flexible privacy-preserving dual authentication scheme for IoT-connected healthcare sensor networks contains seven algorithms, namely Setup, KGenS, KGenA, Sign, SignChg, Verify, and Trace, which are described as follows:

- $\left(msk,mpk\right)\leftarrow \mathrm{Setup}\left(1^{\lambda }\right)$: TA runs the setup algorithm, inputs the security parameter λ, and outputs the public/private key pair (mpk,msk).
- (sk,pk)←KGenS(msk,mpk): TA runs the key generation algorithm for signers, inputs (mpk,msk), and outputs the signer’s signing public/private key pair (pk,sk).
- $\left(sk_{S},sk_{A},pk_{A}\right)\leftarrow \mathrm{KGenA}\left(msk,mpk,S\right)$: TA runs the key generation algorithm for all entities, inputs (mpk,msk) and the entity-owned attribute set *S*, and outputs the private attribute key $sk_{S}$ and the public/private key pair $\left(sk_{A},pk_{A}\right)$ of the entity.
- σ←Sign(mpk,sk,A,m): Users or medical institutions run the signing algorithm, input mpk, the private signing key sk, the access control policy *A*, and the message *m*, and output a valid signature σ.
- $\sigma^{\prime }\leftarrow \mathrm{SignChg}\left(mpk,\sigma ,m^{\prime },sk_{S}\right)$: Authorized entities run the signature modification algorithm, input mpk, the previous signature σ, the modified message $m^{\prime }$, and the private attribute key $sk_{S}$, and output a new signature $\sigma^{\prime }$ for the modified message.
- b←Verify(mpk,σ,m,pk): Anyone can run the verification algorithm with input mpk, σ, *m*, and the signer’s public key pk. The algorithm outputs 1 if the signature is valid; otherwise, it outputs 0.
- $ID\leftarrow \mathrm{Trace}(mpk,\sigma ,\pi_{\mathrm{ABSS}})$: Authorized entities run the trace algorithm with input mpk, σ, and the proof $\pi_{\mathrm{ABSS}}$, and output the identity ID of the last signer.

### 3.4. Security Model

#### 3.4.1. Definition: Fine-Grained Access Control

We consider two adversaries with formal descriptions of the fine-grained access control of signed data. Adversary $A_{1}$ does not own the set of attributes that satisfy the access control policy, while adversary $A_{2}$ attempts to edit the inadmissible parts of the signed data. We introduce two games between the challenger *C* and adversaries $A_{1}$ and $A_{2}$.

For $A_{1}$, challenger *C* generates (msk,mpk) by running $\mathrm{Setup}(1^{\lambda })$, stores msk locally, and sends mpk to $A_{1}$. In the query phase, $A_{1}$ may adaptively issue KGenA, Sign, SignChg, Verify, and Trace queries. In particular, for KGenA queries, $A_{1}$ queries the authorized entity’s key for

((mpk,msk),S)withA(S)=0,

and *C* returns $sk_{S}$ and $\left(pk_{A},sk_{A}\right)$ by running KGenA(msk,mpk,S). For signing and changing queries, *C* returns σ or $\sigma^{\prime }$ by running the corresponding algorithms, and for verification and tracing queries, *C* returns the output of Verify or Trace.

In the challenge phase, $A_{1}$ adaptively selects an unauthorized attribute set *S* such that A(S)=0, generates the challenged signature $\sigma^{*}$ for the challenged message $m^{*}$ by running SignChg, and returns $\left(S,m^{*},\sigma^{*}\right)$ to *C*. In the verify phase, $A_{1}$ makes polynomially many queries and obtains

$$Q={\left\{sk_{S},S,m_{i},A_{i},\sigma_{i}\right\}}_{i=1}^{\left|Q\right|}$$

through *L* queries. Challenger *C* runs $\mathrm{Verify}(mpk,pk,m^{*},\sigma^{*})$ and outputs a bit $b_{0}$. If $b_{0}=1$, *C* checks whether there exists i∈[|Q|] such that $\sigma^{*}$ is accepted while A(S)=0. If such an *i* exists, *C* outputs $b_{1}=1$; otherwise, *C* outputs $b_{1}=0$. The advantage of $A_{1}$ is $\mathrm{Pr}[b_{1}=1]$. If for any polynomial-time adversary $A_{1}$,

$$\mathrm{Pr}\left[b_{1}=1\right]<\frac{1}{\mathrm{poly}(n)}$$

for sufficiently large *n*, the proposed scheme satisfies signature unforgeability against unauthorized modification.

For $A_{2}$, the setup and query phases are the same. In the challenge phase, $A_{2}$ adaptively selects an attribute set *S* such that A(S)=1, and generates the challenged signature $\sigma^{*}$ for the challenged message $m^{*}$ by SignChg, where $m^{*}$ does not include all inadmissible blocks. It sends $\left(S,m^{*},\sigma^{*}\right)$ to *C*. After polynomially many queries, $A_{2}$ obtains

$$Q={\left\{sk_{S},S,m_{i},A_{i},\sigma_{i}\right\}}_{i=1}^{\left|Q\right|}.$$

Challenger *C* runs $\mathrm{Verify}(mpk,pk,A,m^{*},\sigma^{*})$ and outputs $b_{0}$. If $b_{0}=1$, *C* checks whether there exists i∈[|Q|] such that $m^{*}$ does not contain all inadmissible blocks. If so, *C* outputs $b_{1}=1$; otherwise, it outputs $b_{1}=0$. The advantage of $A_{2}$ is $\mathrm{Pr}[b_{1}=1]$. If for any polynomial-time adversary $A_{2}$,

$$\mathrm{Pr}\left[b_{1}=1\right]<\frac{1}{\mathrm{poly}(n)}$$

for sufficiently large *n*, the proposed scheme satisfies the unforgeability of the signature.

#### 3.4.2. Definition: Transparency

We say that a fine-grained and flexible dual authentication scheme for IoT-connected healthcare sensor networks enables transparency if the PPT adversary cannot distinguish, with non-negligible probability, between the signature of the modified data generated by the authorized entity and the signature of the original signer.

#### 3.4.3. Definition: Privacy Preserving

We say a fine-grained and flexible dual authentication scheme for IoT-connected healthcare sensor networks achieves privacy preserving if no PPT adversary can obtain the user’s personal information from the transmitted data.

#### 3.4.4. Definition: Accountability

We say a fine-grained and flexible dual authentication scheme for IoT-connected healthcare sensor networks supports accountability if authorized entities can trace the original signer’s or the latest modifier’s identity of the data from any valid signature with non-negligible probability.

## 4. The Proposed Scheme

### 4.1. Overview

In an IoT-connected healthcare sensor-network deployment, wearable sensors, home gateways, mobile terminals, and edge nodes may relay signed health data from users to medical institutions, support emergency medical communication, and help perform lightweight verification before data are forwarded. Such sensing and edge nodes are useful for continuous monitoring, coverage extension, and mobility, but they should not expose patient identity, medical-institution identity, or the linkage between multiple signatures generated by the same entity. Therefore, the cryptographic layer must simultaneously support privacy-preserving dual authentication, fine-grained modification authorization, and efficient verification.

To realize fine-grained and flexible dual authentication for IoT-connected healthcare sensor networks, we first try out the idea from the recent work P3S [4] to allow the authorized medical institutions to add and remove the user’s personal information for the signed data and generate a new indistinguishable signature for the modified data. However, the direct use of the P3S [4] severely exposes the identities of users and medical institutions. This is due to two ineluctable reasons: (1) the signing key used by the original signer is tied to his/her identity; (2) P3S contains a public key encryption, whose key is also bound to the identity. Therefore, P3S is ill-suited for privacy-preserving healthcare sensor networks, which require users’ and medical institutions’ identities to be hidden during transmission through sensor, edge, and cloud channels. To solve the mentioned problems, we design a new attribute-based sanitizable signature (ABSS) based on Ciphertext-Policy Attribute-Based Encryption (CP-ABE) and Signature with Flexible Public Key (SFPK). The general construction of ABSS is shown in Table 2. In ABSS, the signer (user or medical institutions) signs the data using different keys each time, which belong to the same equivalence class. Only authorized entities with the corresponding trapdoor can link these different keys. Therefore, ABSS supports the privacy preservation of the signer’s identity. ABSS also supports fine-grained access control and accountability like P3S [4]. Encouragingly, ABSS has smaller signature and key lengths and less computational overhead for signing and signature changing compared with the state-of-the-art P3S [4]. Further, we propose a fine-grained and flexible dual authentication scheme for IoT-connected healthcare sensor networks based on the proposed ABSS. In our scheme, the user and the medical institutions sign the data with a fresh related signing key each time and specify who can modify the data and which parts of their signed data can be modified through a specified fine-grained access control policy. The transmitted data contain no personal information even when they pass through sensor gateways, edge nodes, or cloud servers. Authorized medical institutions can add or remove the user’s personal information in the signed data (the transmitted health data or the transmitted diagnosis), and non-interactively generate the new indistinguishable signature for the modified data. Our scheme supports accountability, i.e., authorized entities can trace the original signer and the modifier of the signed data. In addition, the signed health data and diagnoses contain timestamps to ensure accurate treatment and to prevent replay attacks. The following subsections describe the proposed scheme in detail.

### 4.2. Description of the Proposed Scheme

A fine-grained and flexible dual authentication scheme for IoT-connected healthcare sensor networks includes seven algorithms. Table 2 gives the generic construction of ABSS based on CP-ABE, SFPK, NIZK, and PHF.

- **Setup.** $\left(msk,mpk\right)\leftarrow \mathrm{Setup}\left(1^{\lambda }\right)$. Let
  $$\hat{e}:G_{1}\times G_{2}\to G_{T}$$
  be a bilinear pairing, where $G_{1}$, $G_{2}$, and $G_{T}$ are three multiplicative cyclic groups with prime order *p*, and $g_{1}$ and $g_{2}$ are generators of $G_{1}$ and $G_{2}$, respectively. TA randomly chooses
  $$\left(a_{1},a_{2},b_{1},b_{2}\right)\leftarrow Z_{p}^{*},\;\left(d_{1},d_{2},d_{3}\right)\leftarrow Z_{p},$$
  selects a random string ρ, and obtains $crs_{\Omega }\leftarrow {\mathrm{Setup}}_{\Omega }\left(1^{\lambda }\right)$. It then computes
  $$\begin{array}{cccc} H_{1} & =g_{2}^{a_{1}}, & H_{2} & =g_{2}^{a_{2}},\\ T_{1} & =\hat{e}{\left(g_{1},g_{2}\right)}^{d_{1}a_{1}+d_{3}}, & T_{2} & =\hat{e}{\left(g_{1},g_{2}\right)}^{d_{2}a_{2}+d_{3}}. \end{array}$$
  Finally, TA outputs
  $$\begin{array}{cc} mpk & =(g_{2},H_{1},H_{2},T_{1},T_{2}),\\ msk & =(g_{1},g_{2},a_{1},a_{2},b_{1},b_{2},g_{1}^{d_{1}},g_{1}^{d_{2}},g_{1}^{d_{3}}). \end{array}$$
- **KGenS.** (sk,pk)←KGenS(msk,mpk). TA first computes
  $$K_{\mathrm{PHF}}\leftarrow \mathrm{PHF}.\mathrm{Gen}\left(\lambda \right),$$
  randomly selects $A,B,C,D,X\leftarrow G_{1}$ and $z\leftarrow Z_{p}^{*}$, and computes
  $$h\leftarrow \hat{e}\left(X^{z},g_{2}\right).$$
  It outputs
  $$sk=\left(z,X\right),\;pk=\left(A,B,C,D,h,K_{\mathrm{PHF}}\right).$$
- **KGenA.** $\left(sk_{S},sk_{A},pk_{A}\right)\leftarrow \mathrm{KGenA}\left(msk,mpk,S\right)$. TA first selects $r_{1},r_{2}\leftarrow Z_{p}$ and computes
  $$sk_{0}=\left(g_{2}^{b_{1}r_{1}},g_{2}^{b_{2}r_{2}},g_{2}^{r_{1}+r_{2}}\right).$$
  For at∈S and s=1,2, it computes
  $$sk_{at,s}=H{\left(at1s\right)}^{b_{1}r_{1}/a_{s}}\cdot H{\left(at2s\right)}^{b_{2}r_{2}/a_{s}}\cdot H{\left(at3s\right)}^{\left(r_{1}+r_{2}\right)/a_{s}}\cdot g_{1}^{\sigma_{at}/a_{s}},$$
  where $\sigma_{at}\leftarrow Z_{p}$ and $H:{\left\{0,1\right\}}^{*}\to G_{1}$. TA sets
  $$sk_{at}=\left(sk_{at,1},sk_{at,2},g_{1}^{-\sigma_{at}}\right).$$
  For s=1,2, TA computes
  $$sk_{s}^{\prime }=g_{1}^{d_{s}}\cdot H{\left(011s\right)}^{b_{1}r_{1}/a_{s}}\cdot H{\left(012s\right)}^{b_{2}r_{2}/a_{s}}\cdot H{\left(013s\right)}^{\left(r_{1}+r_{2}\right)/a_{s}}\cdot g_{1}^{k/a_{s}},$$
  where $k\leftarrow Z_{p}$, and sets
  $$sk^{\prime }=\left(sk_{1}^{\prime },sk_{2}^{\prime },g_{1}^{d_{3}}\cdot g_{1}^{-k}\right).$$
  The entity computes y=f(x), where *f* is a one-way function and *x* is a random number in $G_{1}$, sends *y* to TA, and proves to TA that it owns *x* by zero knowledge. Finally, TA outputs
  $$sk_{S}=\left(sk_{0},{\left\{sk_{at}\right\}}_{at\in S},sk^{\prime }\right),\;\left(sk_{A}=x,pk_{A}=y\right).$$
- **Sign.** σ←Sign(mpk,sk,A,m). The user or medical institution computes
  $$\delta \leftarrow (d,g_{2}^{y_{2}},g_{2}^{a},g_{2}^{b},g_{2}^{c},\left(g_{2}^{\mu_{0}},g_{2}^{\mu_{1}},\ldots ,g_{2}^{\mu_{\lambda }}\right)),$$
  where ω is a random coin, $a,b,c,d,y_{2}\leftarrow Z_{p}^{*}$, and $\mu_{i}\leftarrow Z_{p}$ for i∈{0,…,λ}. It selects $n\leftarrow Z_{p}^{*}$ and computes
  $$pk^{\prime }=\left(A^{n},B^{n},C^{n},D^{n},h^{n},K_{\mathrm{PHF}}^{n}\right),\;sk^{\prime }=\left(z,X^{n}\right).$$
  Similarly, it selects $n^{\prime }\leftarrow Z_{p}^{*}$ and obtains
  $$pk^{\prime \prime }=\left(A^{n^{\prime }},B^{n^{\prime }},C^{n^{\prime }},D^{n^{\prime }},h^{n^{\prime }},K_{\mathrm{PHF}}^{n^{\prime }}\right),\;sk^{\prime \prime }=\left(z,X^{n^{\prime }}\right).$$
  Then it computes $C_{\mathrm{CP}\text{-}\mathrm{ABE}}\leftarrow \mathrm{CP}\text{-}\mathrm{ABE}.\mathrm{Encrypt}\left(sk_{\mathrm{SFPK}}^{\prime },\delta ,r\right)$. Specifically, it randomly chooses $s_{1},s_{2}\leftarrow Z_{p}$ and computes
  $$ct_{0}=\left(H_{1}^{s_{1}},H_{2}^{s_{2}},g_{2}^{s_{1}+s_{2}}\right),$$
  $$ct_{i,l}=H{\left(f\left(i\right)l1\right)}^{s_{1}}\cdot H{\left(f\left(i\right)l2\right)}^{s_{2}}\cdot \prod_{j=1}^{n_{2}}{\left[H{\left(0jl1\right)}^{s_{1}}\cdot H{\left(0jl2\right)}^{s_{2}}\right]}^{M_{i,j}},$$
  where (M,f) is a monotone span program corresponding to the access structure, *M* has $n_{1}$ rows and $n_{2}$ columns, $i=1,\ldots ,n_{1}$, and l=1,2,3. It computes
  $$ct^{\prime }=T_{1}^{s_{1}}\cdot T_{2}^{s_{2}}\cdot (sk_{\mathrm{SFPK}}^{\prime }∥\delta ∥r),$$
  and outputs
  $$C_{\mathrm{CP}\text{-}\mathrm{ABE}}=\left(ct_{0},ct_{1},\ldots ,ct_{n_{1}},ct^{\prime }\right).$$
  Next, it computes
  $${\mathrm{Sig}}^{1}\leftarrow \mathrm{SFPK}.{\mathrm{Sign}}_{sk^{\prime }}\left(pk^{\prime },pk^{\prime \prime },m,C_{\mathrm{CP}\text{-}\mathrm{ABE}},\pi +r,y_{1}+r\right),$$
  where $\pi \leftarrow {\mathrm{Prove}}_{\Omega }\left(crs_{\Omega },x,st\right)$ and st is the statement. It chooses $r^{\prime }\leftarrow Z_{p}^{*}$ and computes
  $${\mathrm{Sig}}^{1}=\left(X^{y}\cdot {\left(H_{K_{\mathrm{PHF}}}(m∥C_{\mathrm{CP}\text{-}\mathrm{ABE}}∥\pi +r∥y_{1}+r)\right)}^{r^{\prime }},g_{1}^{r^{\prime }},g_{2}^{r^{\prime }}\right).$$
  In the same vein, it computes
  $${\mathrm{Sig}}^{2}\leftarrow \mathrm{SFPK}.{\mathrm{Sign}}_{sk^{\prime \prime }}\left(H\left(i∥U_{A}\right),i∥U_{A},t,y+r,H\left(m\right),A\right),$$
  where $U_{A}$ represents a collection of message blocks that are not allowed to be modified and *t* represents the expiration time of the signature. Finally, it outputs
  $$\sigma =\{C_{\mathrm{CP}\text{-}\mathrm{ABE}},{\mathrm{Sig}}^{1},{\mathrm{Sig}}^{2},A\}.$$
- **SignChg.** $\sigma^{\prime }\leftarrow \mathrm{SignChg}\left(mpk,\sigma ,m^{\prime },sk_{S}\right)$. The authorized entity computes
  $$\left(sk^{\prime },r,\delta \right)\leftarrow \mathrm{CP}\text{-}\mathrm{ABE}.{\mathrm{Decrypt}}_{sk_{\mathrm{CP}\text{-}\mathrm{ABE}}}\left(C_{\mathrm{CP}\text{-}\mathrm{ABE}}\right).$$
  Let $v={\left\{v_{i}\right\}}_{i\in \{1,2,\ldots ,n_{2}\}}$ satisfy M·v=(1,0,…,0). The authorized entity computes
  $$\begin{array}{cc} num= & ct^{\prime }\cdot \hat{e}\left(\prod_{v_{i}\in \left\{1,\ldots ,n_{2}\right\}}ct_{v_{i},1}^{v_{i}},sk_{0,1}\right)\cdot \hat{e}\left(\prod_{v_{i}\in \left\{1,\ldots ,n_{2}\right\}}ct_{v_{i},2}^{v_{i}},sk_{0,2}\right)\\ & \cdot \hat{e}\left(\prod_{v_{i}\in \left\{1,\ldots ,n_{2}\right\}}ct_{v_{i},3}^{v_{i}},sk_{0,3}\right), \end{array}$$
  and
  $$\begin{array}{cc} den= & \hat{e}\left(sk_{1}^{\prime }\cdot \prod_{v_{i}\in \left\{1,\ldots ,n_{2}\right\}}sk_{f(i),1}^{v_{i}},ct_{0,1}\right)\cdot \hat{e}\left(sk_{2}^{\prime }\cdot \prod_{v_{i}\in \left\{1,\ldots ,n_{2}\right\}}sk_{f(i),2}^{v_{i}},ct_{0,2}\right)\\ & \cdot \hat{e}\left(sk_{3}^{\prime }\cdot \prod_{v_{i}\in \left\{1,\ldots ,n_{2}\right\}}sk_{f(i),3}^{v_{i}},ct_{0,3}\right). \end{array}$$
  It outputs
  $$(sk^{\prime },r,\delta )=num/den.$$
  The authorized entity computes
  $${\mathrm{Sig}}^{1\prime }\leftarrow \mathrm{SFPK}.{\mathrm{Sign}}_{sk^{\prime }}\left(pk^{\prime },pk^{\prime \prime },m^{\prime },C_{\mathrm{CP}\text{-}\mathrm{ABE}},\pi^{\prime }+r,y^{\prime }+r\right),$$
  where $y^{\prime }=f\left(x^{\prime }\right)$ and $\pi^{\prime }$ is a proof of $x^{\prime }$. Finally, it outputs
  $$\sigma^{\prime }=\left\{C_{\mathrm{CP}\text{-}\mathrm{ABE}},{\mathrm{Sig}}^{1\prime },{\mathrm{Sig}}^{2},A\right\}.$$
- **Verify.** b←Verify(mpk,σ,m,pk). The verifier first computes H(m) and checks whether it is equal to H(m) in ${\mathrm{Sig}}^{2}$. If so, the verifier checks whether y+r in ${\mathrm{Sig}}^{2}$ is equal to the corresponding value in ${\mathrm{Sig}}^{1}$. If the checks pass, it returns
  $$b\leftarrow \mathrm{SFPK}.\mathrm{Verify}(pk^{\prime },pk^{\prime \prime },m,\sigma );$$
  otherwise, it returns ⊥. If H(m) is not equal to H(m) in ${\mathrm{Sig}}^{2}$, the verifier computes $H(i∥U_{A})$ and checks whether it equals $H(i∥U_{A})$ in ${\mathrm{Sig}}^{2}$, and then checks whether y+r in ${\mathrm{Sig}}^{2}$ equals the corresponding value in ${\mathrm{Sig}}^{1}$. If all checks pass, it returns *b*; otherwise, it returns ⊥.
- **Trace.** $ID\leftarrow \mathrm{Trace}(mpk,\sigma ,\pi_{\mathrm{ABSS}})$. If
  $$1\leftarrow {\mathrm{Verify}}_{\Omega }\left(crs_{\Omega },st,\pi \right),$$
  the algorithm returns the identity ID corresponding to *y*; otherwise, it returns ⊥.

### 4.3. A Verifiable Outsourced Authentication Scheme

To improve the clarity of the contribution, we further clarify why the proposed ABSS-based dual authentication scheme is not a direct listing of existing primitives. Previous sanitizable signatures can support controlled modification, and previous attribute-based signatures can support expressive authorization, but they do not jointly solve the following healthcare sensor-network requirements: signer-identity hiding without storing many pseudonyms, fine-grained authorization of which entity may modify which signed blocks, accountability for malicious signers or modifiers, and lightweight online verification at wearable sensors, sensor gateways, or mobile terminals. P3S is the closest baseline, but its signing key and public-key encryption component are identity-bound. In contrast, our construction uses SFPK to generate fresh related signing keys in the same equivalence class for each signing event, so different signatures cannot be linked by unauthorized entities. CP-ABE is then used as the policy-enforcement layer that releases the sanitizing capability only to entities whose attributes satisfy the specified access policy. The new component is therefore the integration of SFPK-based unlinkable signing, CP-ABE-based sanitizer authorization, and sanitizable dual authentication into one healthcare workflow with outsourced verification.

For reproducibility, the ABSS workflow is made explicit through the seven algorithms described above. In the system initialization phase, TA runs Setup to generate (mpk,msk), then runs KGenS for users and medical institutions and KGenA for authorized entities with attribute set *S*. In the signing phase, a user or medical institution encodes the access policy *A* as a monotone span program (M,f), signs the health data or diagnosis with a fresh SFPK-related key, and encrypts the temporary signing capability under the CP-ABE policy. In the sanitization phase, only an entity whose attributes satisfy *A* can compute a reconstruction vector *v* with M·v=(1,0,…,0), decrypt the temporary signing capability, modify only the admissible data blocks, and generate an indistinguishable updated signature by SignChg. In the verification and tracing phases, Verify checks the hash of the whole message or the protected inadmissible blocks, and Trace recovers the responsible signer or modifier only for authorized accountability.

We also clarify the attack coverage. If a sensor gateway or edge relay is compromised, it can observe and forward signed traffic but cannot recover signer identity or generate a valid modified signature without satisfying the CP-ABE policy. If one cloud server in outsourced verification is malicious, the randomized blinding values and the use of two mutually distrusted servers prevent it from learning the underlying pairing inputs; incorrect outsourced results are detected by the final verification equation. Collusion between authorized modifiers does not permit modification of inadmissible blocks, because ${\mathrm{Sig}}^{2}$ binds the protected block digest $H(i∥U_{A})$ to the signature. Insider attacks by authorized entities are handled through accountability: a valid modified signature still allows authorized tracing of the latest signer or modifier. Replay attacks are mitigated because each signature includes the expiration timestamp *t*, and stale signatures are rejected during verification.

The deployment process in a real IoT healthcare sensor network is as follows. Wearable sensors or mobile terminals collect health data and remove explicit identifiers before transmission. A sensor gateway or edge relay forwards the signed data and may invoke outsourced verification when local resources are limited. Authorized medical institutions use their attribute keys to recover the necessary personal context, add or remove only permitted fields, generate an updated sanitizable signature, and return a diagnosis. Users, family members, and other authorized institutions can then verify the diagnosis source without learning unnecessary institutional or patient identity information. This workflow separates data forwarding, signature verification, controlled modification, and traceability, which is consistent with practical sensor-edge-cloud healthcare deployments.

**Figure 3:**
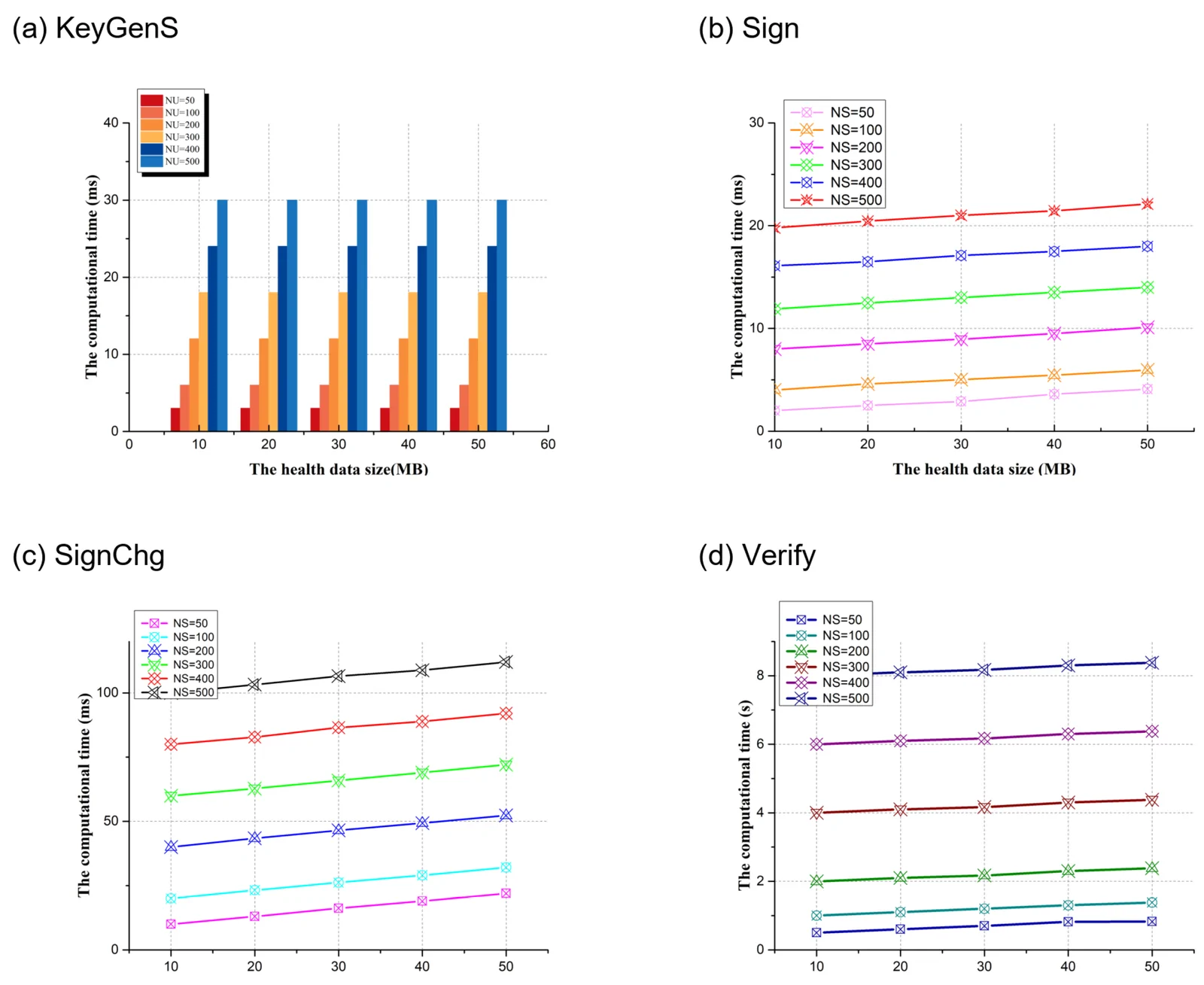
The computational time of the proposed scheme in the KeyGenS, Sign, SignChg and Verify phases.

**Figure 4:**
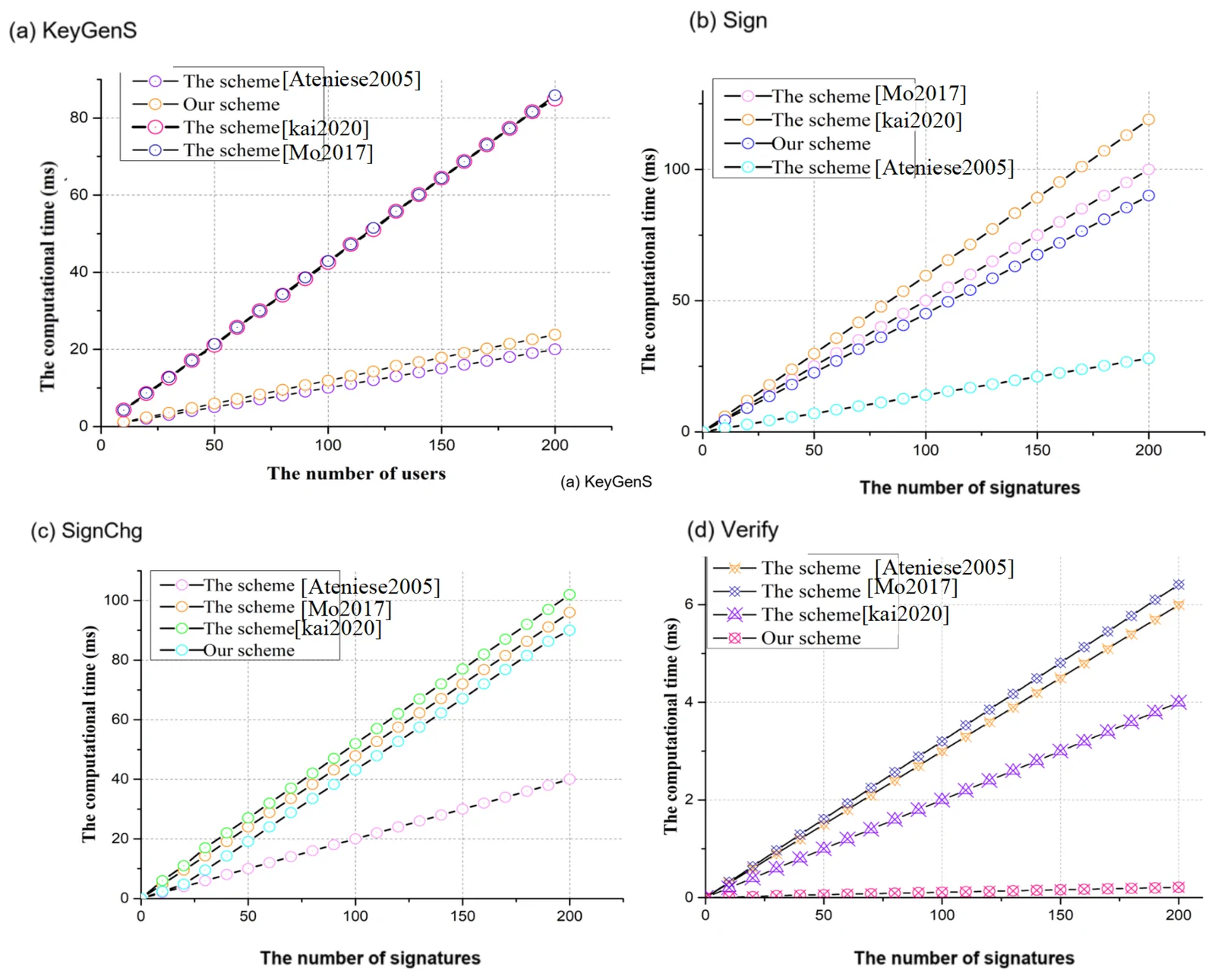
Performance comparison between the proposed scheme and related schemes in the KeyGenS, Sign, SignChg and Verify phases [4,5,23].

Finally, the experimental comparison is clarified in terms of verification time, computation cost, storage/communication overhead, and security guarantees. Storage and communication overhead are reflected by the signing-key, authorized-entity-key, and signature lengths in Table 1. Computation overhead is measured for KGenS, Sign, SignChg, and Verify in Figure 3 and Figure 4. The implementation uses C++ with Pairing-Based Cryptography (PBC) 0.5.14 and GNU Multiple Precision Arithmetic (GMP), an Intel Core i5-4300 CPU at 2.13 GHz with 8.0 GB RAM, and 160-bit elements in $Z_{p}^{*}$; the same implementation environment and parameter setting are used for the compared schemes. The security comparison follows the formal properties defined in Section 3: fine-grained access control, unforgeability, transparency, privacy preserving, accountability, and friendliness to resource-constrained users.

The most time-consuming operation in the proposed scheme is the pairing operation in the Verify algorithm. To make the scheme more user-friendly for resource-constrained entities, we present a verifiable outsourced authentication scheme that retains the advantages of the above scheme while allowing time-consuming pairing operations to be outsourced to two mutually distrusted cloud servers [29]. The verifiable outsourced authentication scheme differs only in the following two algorithms.

- **Pre-computation.** τ←PreC(q) is performed offline by the entity. The entity randomly selects *q* integers $\alpha_{1},\ldots ,\alpha_{q}\in Z_{p}$ and computes
  $$\beta_{j1}=\alpha_{j}g_{1},\;\beta_{j2}=\alpha_{j}g_{2},\;j=1,\ldots ,q.$$
  It computes $\hat{e}\left(g_{1},g_{2}\right)$ and stores $\hat{e}\left(g_{1},g_{2}\right)$ and ${\left\{\alpha_{j},\beta_{j1},\beta_{j2}\right\}}_{j=1,\ldots ,q}$ locally. For vector generation, the entity selects a set *E* such that |E|=k and E⊆{1,…,q}. For each j∈E, it randomly chooses $\chi_{j}\in \left\{1,\ldots ,v-1\right\}$, where *v* is a small integer and v>1, and computes
  $$x_{1}=\sum_{j\in E}\alpha_{j}\chi_{j}\;\mathrm{mod}\;p.$$
  If $x_{1}=0\;\mathrm{mod}\;p$, it repeats the operation; otherwise, it computes
  $$x_{1}g_{1}=\sum_{j\in E}\chi_{j}\beta_{j1}.$$
  In the same vein, it computes $\left(x_{3},x_{3}g_{1}\right)$, $\left(x_{4},x_{4}g_{2}\right)$, $\left(x_{7},x_{7}g_{1}\right)$, and $\left(x_{8},x_{8}g_{2}\right)$. It randomly selects $x_{2},x_{5},x_{6}\leftarrow Z_{p}^{*}$ and computes
  $$x_{1}^{-1}x_{2}g_{2},\;x_{1}x_{2}^{-1}x_{5}g_{1},\;x_{1}^{-1}x_{6}g_{2},\;\hat{e}{\left(g_{1},g_{2}\right)}^{x_{7}x_{8}},\;\hat{e}{\left(g_{1},g_{2}\right)}^{x_{5}+x_{6}-x_{2}}.$$
  Finally, it stores
  $$\begin{array}{cc} \tau = & (x_{1}g_{1},x_{3}g_{1},x_{7}g_{1},x_{1}^{-1}x_{2}g_{2},x_{1}x_{2}^{-1}x_{5}g_{1},x_{4}g_{2},\\ & x_{1}^{-1}x_{6}g_{2},\hat{e}{\left(g_{1},g_{2}\right)}^{x_{7}x_{8}},\hat{e}{\left(g_{1},g_{2}\right)}^{x_{5}+x_{6}-x_{2}},x_{8}g_{2}). \end{array}$$
- **Outsourced verification.** b←Verify(mpk,σ,m,pk,τ). When the verifier runs $\mathrm{SFPK}.\mathrm{Verify}(pk^{\prime },pk^{\prime \prime },m,\sigma )$, the pairing operations can be outsourced to two cloud servers $U_{1}$ and $U_{2}$. Let
  $${\mathrm{Sig}}^{1}=\left(X^{z}\cdot {\left(H_{K_{\mathrm{PHF}}}\left(m\right)\right)}^{n},g_{1}^{n},g_{2}^{n}\right).$$
  The verifier needs to check
  $$\hat{e}\left(g_{1}^{n},g_{2}\right)=\hat{e}\left(g_{1},g_{2}^{n}\right).$$
  To outsource $\hat{e}\left(g_{1}^{n},g_{2}\right)$, the verifier sends
  $$g_{1}^{n}+x_{1}g_{1},\;g_{2}+x_{1}^{-1}x_{2}g_{2},\;x_{3}g_{1},\;x_{4}g_{2}$$
  to $U_{1}$. Then $U_{1}$ computes
  $$\alpha_{1}=\hat{e}\left(g_{1}^{n}+x_{1}g_{1},g_{2}+x_{1}^{-1}x_{2}g_{2}\right),\;\alpha_{2}=\hat{e}\left(x_{3}g_{1},x_{4}g_{2}\right),$$
  and sends $\alpha_{1}$ and $\alpha_{2}$ to the verifier. The verifier sends
  $$\begin{array}{c} g_{1}^{n}+x_{1}x_{2}^{-1}x_{5}g_{1},\;-x_{1}^{-1}x_{2}g_{2},\;-x_{1}g_{1},\;g_{2}+x_{1}^{-1}x_{6}g_{2},\\ x_{3}g_{1},\;x_{4}g_{2},\;x_{7}g_{1},\;x_{8}g_{2} \end{array}$$
  to $U_{2}$. Then $U_{2}$ computes
  $$\begin{array}{cc} \alpha_{1}^{\prime } & =\hat{e}\left(g_{1}^{n}+x_{1}x_{2}^{-1}x_{5}g_{1},-x_{1}^{-1}x_{2}g_{2}\right),\\ \alpha_{2}^{\prime } & =\hat{e}\left(-x_{1}g_{1},g_{2}+x_{1}^{-1}x_{6}g_{2}\right),\\ \alpha_{3}^{\prime } & =\hat{e}\left(x_{3}g_{1},x_{4}g_{2}\right),\\ \alpha_{4}^{\prime } & =\hat{e}\left(x_{7}g_{1},x_{8}g_{2}\right), \end{array}$$
  and sends $\left(\alpha_{1}^{\prime },\alpha_{2}^{\prime },\alpha_{3}^{\prime },\alpha_{4}^{\prime }\right)$ to the verifier. The verifier checks whether
  $$\alpha_{2}=\alpha_{3}^{\prime },\;\hat{e}{\left(g_{1},g_{2}\right)}^{x_{7}x_{8}}=\alpha_{4}^{\prime }.$$
  If the checks hold, the verifier sets
  $$\hat{e}\left(g_{1}^{n},g_{2}\right)=\alpha_{1}\alpha_{1}^{\prime }\alpha_{2}^{\prime }\hat{e}{\left(g_{1},g_{2}\right)}^{x_{5}+x_{6}-x_{2}}.$$
  In the same vein, the verifier obtains $\hat{e}\left(g_{1},g_{2}^{n}\right)$ and checks whether
  $$\hat{e}\left(g_{1}^{n},g_{2}\right)=\hat{e}\left(g_{1},g_{2}^{n}\right)$$
  holds. It also checks whether
  $$\hat{e}\left(X^{z}\cdot {\left(H_{K_{\mathrm{PHF}}}\left(m\right)\right)}^{n},g_{2}\right)=h\cdot \hat{e}\left({\left(H_{K_{\mathrm{PHF}}}\left(m\right)\right)}^{n},g_{2}^{n}\right)$$
  holds. If the above equations hold, the verifier outputs 1; otherwise, it outputs 0. Therefore, after outsourcing one pairing operation, the verifier only needs to perform three modular multiplication operations, and the whole verification process only needs six multiplication operations.

## 5. Results

### 5.1. Security Analysis

In this section, we analyze the security of the proposed healthcare sensor-network authentication scheme in terms of fine-grained access control, transparency, privacy preserving, and accountability.

**Theorem** **1** (Fine-Grained Access Control)**.** *For two PPT adversaries $A_{1}$ and $A_{2}$, it is computationally infeasible to generate a valid signature for the modified data. Here, $A_{1}$ represents unauthorized entities that possess an attribute set S such that A(S)=0, and $A_{2}$ updates the parts of the signed data that are not allowed to be modified.*

**Proof.**  To prove this theorem, we define two games between a challenger *C* and PPT adversaries $A_{1}$ and $A_{2}$.

Game 1

The setup and query phases are as in the security model. Next, $A_{1}$ adaptively chooses an unauthorized entity’s attribute set *S* such that A(S)=0. $A_{1}$ attempts to generate the challenged signature $\sigma^{*}$ for the modified data $m^{*}$ by running SignChg. Finally, $A_{1}$ sends $\left(S,m^{*},\sigma^{*}\right)$ to *C*.

Analysis

Assume that $A_{1}$ wins Game 1 with non-negligible probability, i.e., the challenged signature $\sigma^{*}$ passes the Verify algorithm. According to the security of SFPK [30], $A_{1}$ must know the private signing key $sk^{\prime }$ if it wants to generate a valid signature pair for modified data. This means that $A_{1}$ can correctly decrypt the ciphertext $C_{\mathrm{CP}\text{-}\mathrm{ABE}}$ to obtain $sk^{\prime }$. In other words, an unauthorized entity with attribute set *S* and A(S)=0 can correctly decrypt the ciphertext, which contradicts the security of CP-ABE [28]. Therefore, it is computationally infeasible for $A_{1}$ to generate a valid signature for the modified data.

Game 2

The setup and query phases are again as in the security model. Next, $A_{2}$ adaptively chooses an entity’s attribute set *S* such that A(S)=1. $A_{2}$ attempts to generate the challenged signature $\sigma^{*}$ for the modified data $m^{*}$ by running SignChg. The modified data $m^{*}$ does not contain all inadmissible blocks. Finally, $A_{2}$ sends $\left(S,m^{*},\sigma^{*}\right)$ to *C*.

Analysis

Assume that $A_{2}$ wins Game 2 with non-negligible probability, i.e., the challenged signature $\sigma^{*}$ passes the Verify algorithm. In Verify, the challenger computes $H(i∥U_{A})$ and checks whether it equals the corresponding $H(i∥U_{A})$ in ${\mathrm{Sig}}^{2}$. Thus, $A_{2}$ needs to forge a valid signature

$${\mathrm{Sig}}^{2\prime }\leftarrow \mathrm{SFPK}.{\mathrm{Sign}}_{sk^{\prime \prime }}\left(H^{\prime }\left(i^{\prime }∥{U\prime }_{A}\right),i^{\prime }∥{U\prime }_{A},t,y+r,H\left(m\right),A\right)$$

for the modified data $m^{*}$. Because $m^{*}$ does not contain all inadmissible blocks, the security of SFPK [30] implies that the probability of forging ${\mathrm{Sig}}^{2\prime }$ without the private signing key $sk^{\prime \prime }$ is negligible. Therefore, it is computationally infeasible for $A_{2}$ to generate a valid signature for the modified data. Hence, the proposed scheme achieves fine-grained access control. □

**Theorem** **2** (Transparency)**.** *For any PPT adversary, it is computationally infeasible to distinguish the modified signature from the original signature.*

**Proof.**  The original signature σ and the signature $\sigma^{\prime }$ for the modified data are

$$\sigma =\left\{\begin{array}{c} A;\\ C_{\mathrm{CP}\text{-}\mathrm{ABE}}\leftarrow \mathrm{CP}\text{-}\mathrm{ABE}.\mathrm{Encrypt}\left(sk^{\prime },\delta ,r\right);\\ {\mathrm{Sig}}^{1}\leftarrow \mathrm{SFPK}.{\mathrm{Sign}}_{sk^{\prime }}\left(pk^{\prime },pk^{\prime \prime },m,C_{\mathrm{CP}\text{-}\mathrm{ABE}},\pi +r,y+r\right);\\ {\mathrm{Sig}}^{2}\leftarrow \mathrm{SFPK}.{\mathrm{Sign}}_{sk^{\prime \prime }}\left(H\left(i∥U_{A}\right),i∥U_{A},t,y+r,H\left(m\right),A\right), \end{array}\right.$$

and

$$\sigma^{\prime }=\left\{\begin{array}{c} A;\\ C_{\mathrm{CP}\text{-}\mathrm{ABE}}\leftarrow \mathrm{CP}\text{-}\mathrm{ABE}.\mathrm{Encrypt}\left(sk^{\prime },\delta ,r\right);\\ {\mathrm{Sig}}^{1\prime }\leftarrow \mathrm{SFPK}.{\mathrm{Sign}}_{sk^{\prime }}\left(pk^{\prime },pk^{\prime \prime },m^{\prime },C_{\mathrm{CP}\text{-}\mathrm{ABE}},\pi^{\prime }+r,y^{\prime }+r\right);\\ {\mathrm{Sig}}^{2}\leftarrow \mathrm{SFPK}.{\mathrm{Sign}}_{sk^{\prime \prime }}\left(H\left(i∥U_{A}\right),i∥U_{A},t,y+r,H\left(m\right),A\right). \end{array}\right.$$

The only difference between σ and $\sigma^{\prime }$ is that ${\mathrm{Sig}}^{1}$ and ${\mathrm{Sig}}^{1\prime }$ have different values. However, ${\mathrm{Sig}}^{1}$ and ${\mathrm{Sig}}^{1\prime }$ are both outputs of $\mathrm{SFPK}.{\mathrm{Sign}}_{sk^{\prime }}\left(\cdot \right)$ and belong to the same distribution. Therefore, for any PPT adversary, it is computationally infeasible to distinguish the signature of modified data from the signature of original data, and the scheme achieves transparency in healthcare sensor-network data transmission. □

**Theorem** **3** (Privacy Preserving)**.** *In the proposed scheme, the transmitted data contains no personal information of users or medical institutions.*

**Proof.**  The user removes personal information before signing and uploading health data, so the transmitted health data contains no user’s personal information. In the Sign and SignChg algorithms, the user or medical institution uses a fresh signing key each time. Thus, no one can trace the signer or modifier from the original signature and the modified-data signature except authorized entities. Authorized medical institutions also remove the user’s personal information from the signed diagnosis before uploading it. Therefore, the signed diagnosis contains no user’s personal information, and the scheme achieves privacy preservation. □

**Theorem** **4** (Accountability)**.** *In the proposed scheme, only authorized entities can extract the last modifier’s identity from any valid signature, and the original signer cannot accuse the authorized entities, or vice versa, of signing.*

**Proof.**  The signature contains the blinded proof π+r. Only authorized entities can correctly decrypt

$$C_{\mathrm{CP}\text{-}\mathrm{ABE}}=\mathrm{CP}\text{-}\mathrm{ABE}.\mathrm{Encrypt}\left(sk^{\prime },\delta ,r\right)$$

to obtain the blinding factor *r*. Thus, authorized entities can recover

π=(π+r)+(−r),y=(y+r)+(−r).

Because *y* corresponds to the entity identity, authorized entities can obtain the identity of the signer or modifier. To prevent authorized entities from impersonating the original signer, the verifier first computes H(m) in Verify and checks whether it equals H(m) in ${\mathrm{Sig}}^{2}$. If they are not equal, the data has been modified. The verifier then checks whether y+r in ${\mathrm{Sig}}^{2}$ equals the corresponding value in ${\mathrm{Sig}}^{1}$. If they are equal, the modifier is trying to impersonate the original signer, so the verifier returns ⊥. Therefore, only authorized entities can extract the last modifier’s identity from any valid signature, and neither party can falsely accuse the other of signing. □

### 5.2. Performance Evaluation

In this section, we first compare the functionality of the proposed scheme with several related schemes, and then perform an experimental analysis of the computational burden of our scheme and the related schemes [4,5,23].

### 5.3. Functionality Comparison

We compare the functionality of the proposed scheme with the related schemes [4,6,9,16,17,21,22,23] As shown in Table 3, only our scheme satisfies all the following properties: fine-grained access control, unforgeability, transparency, accountability, privacy preserving and friendliness to resource-constrained users. The remaining schemes are all unfriendly to resource-constrained users; schemes [16,17,22] do not support transparency, and, apart from our scheme, only the scheme in [4] supports accountability.

### 5.4. Performance Analysis and Comparison

#### 5.4.1. The Comparison of Storage Space

We define the following notations for the comparison. Let L(·) denote the length of the input element. Let $N=p_{1}\cdot p_{2}$, where $p_{1}$ and $p_{2}$ are two prime numbers. Let $e_{1}$ be a random number such that $e_{1}=1\;\mathrm{mod}\;\left(p_{1}-1\right)\left(p_{2}-1\right)$, and let $e_{2}$ be a number such that $e_{1}\cdot e_{2}=1\;\mathrm{mod}\;\left(p_{1}-1\right)\left(p_{2}-1\right)$. Let $n_{1}$ and $n_{2}$ denote the row and column numbers of the monotonic span matrix of the access control policy *A*, respectively. Let $sk_{DS}$ and $S_{DS}$ denote the private signing key and the signature of the standard digital signature scheme.

The concrete instantiation of the standard digital signature is not given in [4]. Here, *p* is the large prime number used in [4] and π is a proof. Finally, |S| denotes the number of attributes in the attribute set *S*. As shown in Table 4, the scheme in [5] has the shortest signing-key length, while our scheme has the shortest signature length. Schemes in [5,23] cannot support fine-grained access control. Our scheme has a smaller key length and signature length compared with the scheme in [4], since $L\left(S_{DS}\right)+L\left(p\right)+L\left(\pi \right)\geq 2L\left(G\right)$ generally. Hence, in terms of storage space overhead, our scheme is efficient.

#### 5.4.2. The Comparison of Computation Overhead

In this subsection, we set the number of users from 50 to 500 and the health-data size from 0 to 50 MB, and conduct the performance evaluation. We further compare our scheme with the schemes in [4,5,23]. The simulation experiments were written in C++ with Pairing-based Cryptography (PBC) version 0.5.14 [31] and GNU Multiple Precision Arithmetic (GMP) [32]. The desktop was equipped with an Intel Core (TM) i5-4300 CPU @ 2.13 GHz and 8.0 GB RAM, and the size of elements in $Z_{p}^{*}$ was 160 bits. The KGenS, Sign, SignChg, and Verify phases were evaluated while the health-data size ranged from 0 to 50 MB and $N_{U}$ ranged from 50 to 500, where $N_{U}$ denotes the number of users. When $N_{U}$ ranges from 50 to 500, the computational time of KGenS has a linear relationship with $N_{U}$ and is independent of the health-data size, as shown in Figure 3. Let $N_{S}$ denote the number of signatures. When $N_{S}$ ranges from 50 to 500, the computational times of Sign, SignChg, and Verify also have linear relationships with $N_{S}$, as shown in Figure 3. For the performance comparison, Figure 4 shows that, when $N_{S}$ ranges from 0 to 200, the KGenS algorithms of the schemes in [4,23] vary from 0 ms to 88.59 ms, while [5] varies from 0 ms to 20.45 ms, and our scheme varies from 0 ms to 22.34 ms. The Sign algorithms of [4,23] vary from 0 ms to 0.128 s and 0.119 s, respectively, while [5] varies from 0 ms to 0.025 s. For SignChg, the schemes in [4,23] vary from 0 ms to 0.109 s and 0.096 s, respectively, while [5] and our scheme vary from 0 ms to 0.042 s and 0 ms to 0.089 s, respectively. For Verify, the schemes in [5,23] vary from 0 ms to 8.04 ms and 6.02 ms, respectively, [4] varies from 0 ms to 4.32 ms, and the proposed scheme takes the shortest time because it outsources the time-consuming pairing operations to two cloud servers. As a result, the verifier only needs to perform three multiplication operations for each pairing operation, which is particularly useful when verification is performed by sensor gateways, mobile edge devices, or wearable healthcare terminals with limited computation and energy budgets.

## 6. Conclusions

In this paper, we proposed a fine-grained and flexible dual authentication scheme for IoT-connected healthcare sensor networks. The proposed scheme supports fine-grained access control, allowing users and medical institutions to specify who can modify signed data and which parts may be modified through a fine-grained access control policy. The scheme also supports privacy preservation and accountability: transmitted healthcare data contain no personal information, and only authorized entities can trace the modifier and the original signer of signed data. These properties are important when health data and diagnoses are forwarded through wearable sensors, sensor gateways, edge nodes, cloud servers, and multiple medical institutions. To further reduce the computational overhead for resource-constrained sensor and IoT participants, we proposed a verifiable outsourced authentication scheme that outsources expensive pairing operations to two mutually distrusted cloud servers. In this way, the verification process only needs to perform six multiplication operations. Therefore, the proposed scheme provides a lightweight privacy-preserving authentication layer for trustworthy healthcare sensing and medical data exchange.

## Figures and Tables

**Table 1:** Comparison between our scheme and the scheme in [4].

Metric	The Scheme in [4]	Ours
Length of signing key	$6L\left(Z_{p}^{*}\right)+4L\left(G\right)+L\left(sk_{DS}\right)$	$L\left(Z_{p}^{*}\right)+3L\left(G\right)$
Length of authorized entities’ key	$3\left(|S|+2\right)L\left(G\right)+L\left(Z_{p}^{*}\right)$	$3\left(|S|+2\right)L\left(G\right)+L\left(Z_{p}^{*}\right)$
Length of signature	$4L\left(Z_{p}^{*}\right)+\left(3n_{1}+5\right)L\left(G\right)+L\left(S_{DS}\right)+L\left(p\right)+L\left(\pi \right)$	$3L\left(Z_{p}^{*}\right)+\left(3n_{1}+7\right)L\left(G\right)$
Time of signature generating ($N_{S}=200$)	0.128 s	0.806 s
Time of signature changing ($N_{S}=200$)	0.109 s	0.089 s

*Notes:* Let L(·) denote the length of the input element. Let $n_{1}$ and $n_{2}$ denote the row and column numbers of the monotonic span matrix of the access control policy *A*, respectively. $S_{DS}$ denotes the signature of the standard digital signature scheme. The concrete instantiation of the standard digital signature is not given in [4]. *p* is the large prime number used in [4] and π is a proof. Finally, |S| denotes the number of attributes in the attribute set *S*. $N_{S}$ denotes the number of signatures.

**Table 2:** The generic construction.

Algorithm	Construction Summary
Setup	$\left(pp_{\mathrm{CP}\text{-}\mathrm{ABE}},msk_{\mathrm{CP}\text{-}\mathrm{ABE}}\right)\leftarrow \mathrm{CP}\text{-}\mathrm{ABE}.\mathrm{Setup}\left(1^{\lambda }\right)$; $\rho \leftarrow \mathrm{SFPK}.\mathrm{Setup}(1^{\lambda })$; $crs_{\Omega }\leftarrow {\mathrm{Setup}}_{\Omega }\left(1^{\lambda }\right)$; return $mpk=\{pp_{\mathrm{CP}\text{-}\mathrm{ABE}},\rho ,crs_{\Omega }\}$ and $msk=msk_{\mathrm{CP}\text{-}\mathrm{ABE}}$.
KGenS	$\left(sk_{\mathrm{SFPK}},pk_{\mathrm{SFPK}}\right)\leftarrow \mathrm{SFPK}.\mathrm{KGen}\left(1^{\lambda },\omega \right)$; return $sk=sk_{\mathrm{SFPK}}$ and $pk=pk_{\mathrm{SFPK}}$.
KGenA	$sk_{\mathrm{CP}\text{-}\mathrm{ABE}}\leftarrow \mathrm{CP}\text{-}\mathrm{ABE}.\mathrm{KeyGen}\left(msk,S\right)$; y=f(x); return $sk_{S}=sk_{\mathrm{CP}\text{-}\mathrm{ABE}}$, $sk_{A}=x$, and $pk_{A}=y$.
Sign	$\delta \leftarrow \mathrm{SFPK}.\mathrm{TKGen}(1^{\lambda },\omega )$; $\left(sk^{\prime },pk^{\prime }\right)\leftarrow \mathrm{SFPK}.\mathrm{ChgKeys}\left(1^{\lambda },n\right)$; $\left(sk^{\prime \prime },pk^{\prime \prime }\right)\leftarrow \mathrm{SFPK}.\mathrm{ChgKeys}\left(1^{\lambda },n^{\prime }\right)$; $C_{\mathrm{CP}\text{-}\mathrm{ABE}}\leftarrow \mathrm{CP}\text{-}\mathrm{ABE}.\mathrm{Encrypt}\left(sk^{\prime },\delta ,r\right)$; ${\mathrm{Sig}}^{1}\leftarrow \mathrm{SFPK}.{\mathrm{Sign}}_{sk^{\prime }}\left(pk^{\prime },pk^{\prime \prime },m,C_{\mathrm{CP}\text{-}\mathrm{ABE}},\pi +r,y+r\right)$; ${\mathrm{Sig}}^{2}\leftarrow \mathrm{SFPK}.{\mathrm{Sign}}_{sk^{\prime \prime }}\left(H\left(i∥U_{A}\right),i∥U_{A},t,y+r,H\left(m\right),A\right)$; return $\sigma =\{C_{\mathrm{CP}\text{-}\mathrm{ABE}},{\mathrm{Sig}}^{1},{\mathrm{Sig}}^{2},A\}$.
SignChg	$\left(sk^{\prime },r,\delta \right)\leftarrow \mathrm{CP}\text{-}\mathrm{ABE}.{\mathrm{Decrypt}}_{sk_{\mathrm{CP}\text{-}\mathrm{ABE}}}\left(C_{\mathrm{CP}\text{-}\mathrm{ABE}}\right)$; ${\mathrm{Sig}}^{1\prime }\leftarrow \mathrm{SFPK}.{\mathrm{Sign}}_{sk^{\prime }}\left(pk^{\prime },pk^{\prime \prime },m^{\prime },C_{\mathrm{CP}\text{-}\mathrm{ABE}},\pi^{\prime }+r,y^{\prime }+r\right)$; $y^{\prime }=f\left(x^{\prime }\right)$; return $\sigma^{\prime }=\left\{C_{\mathrm{CP}\text{-}\mathrm{ABE}},{\mathrm{Sig}}^{1\prime },{\mathrm{Sig}}^{2},A\right\}$.
Verify	$b\leftarrow \mathrm{SFPK}.\mathrm{Verify}(pk_{\mathrm{SFPK}},m,\sigma )$; return *b*.
Trace	If $1\leftarrow {\mathrm{Verify}}_{\Omega }\left(crs_{\Omega },st,\pi \right)$, return ID; if $0\leftarrow {\mathrm{Verify}}_{\Omega }\left(crs_{\Omega },st,\pi \right)$, return ⊥.

**Table 3:** Comparison of functionality among our scheme and related schemes.

Scheme	FGAC	Unforgeability	Transparency	Accountability	Privacy	RCU-Friendly
[6]	N	Y	Y	N	N	N
[9]	N	Y	Y	N	N	N
[16]	N	Y	N	N	N	N
[17]	N	Y	N	N	N	N
[21]	N	Y	Y	N	N	N
[22]	Y	Y	–	–	N	N
[23]	Y	Y	Y	N	Y	N
[4]	Y	Y	Y	Y	Y	N
Ours	Y	Y	Y	Y	Y	Y

FGAC: fine-grained access control; RCU-friendly: friendly to resource-constrained users. N denotes “No”, and Y denotes “Yes”.

**Table 4:** Comparison of storage space between our scheme and related schemes.

Scheme	Length of Signing Key	Length of Authorized Entities’ Key	Length of Signature
[5]	$L\left(N\right)+L\left(e_{1}\right)+L\left(e_{2}\right)$	–	L(m)L(G)
[23]	(2+|S|)L(G)	–	$\left(2+n_{1}+n_{2}\right)L\left(G\right)$
[4]	$6L\left(Z_{p}^{*}\right)+4L\left(G\right)+L\left(sk_{DS}\right)$	$3\left(|S|+2\right)L\left(G\right)+L\left(Z_{p}^{*}\right)$	$4L\left(Z_{p}^{*}\right)+\left(3n_{1}+5\right)L\left(G\right)+L\left(S_{DS}\right)+L\left(p\right)+L\left(\pi \right)$
Ours	$L\left(Z_{p}^{*}\right)+3L\left(G\right)$	$3\left(|S|+2\right)L\left(G\right)+L\left(Z_{p}^{*}\right)$	$3L\left(Z_{p}^{*}\right)+\left(3n_{1}+7\right)L\left(G\right)$

## Data Availability

The datasets presented in this article are not readily available because some algorithms and data are related to our ongoing research. Requests to access the datasets should be directed to hyhou01@163.com.

## Abbreviations

The following abbreviations are used in this manuscript:

ABSS	Attribute-based sanitizable signature.
CP-ABE	Ciphertext-policy attribute-based encryption.
IoT	Internet of Things.
NIZK	Non-interactive zero-knowledge proof.
PBC	Pairing-based cryptography.
PHF	Programmable hash function.
SFPK	Signature with flexible public key.
TA	Trusted authority.
